# Advancing Personalized Intrathecal Therapy: A Quasi-Experimental Study for the Evaluation of Patient Satisfaction and Pain in Ultrasound-Guided Versus Template-Guided Refill Techniques

**DOI:** 10.3390/jpm16050270

**Published:** 2026-05-18

**Authors:** Beatriz Lechuga Carrasco, Beatriz Piqueras-Sola, Nicolás Cordero Tous, Jonathan Cortés-Martín, Juan Carlos Sánchez-García, Raquel Rodríguez-Blanque, Rafael Gálvez Mateos

**Affiliations:** 1 Virgen de las Nieves University Hospital, 18014 Granada, Spain; beatrizlcarrasco@gmail.com (B.L.C.); bpiquerassola@gmail.com (B.P.-S.); nicolas.cordero.sspa@juntadeandalucia.es (N.C.T.); rafaelgalvez@hotmail.com (R.G.M.); 2 Department of Nursing, Faculty of Health Sciences, University of Granada, 18012 Granada, Spain; jsangar@ugr.es (J.C.S.-G.); rarobladoc@ugr.es (R.R.-B.)

**Keywords:** intrathecal infusion pump, pain, ultrasound, satisfaction, personalized medicine

## Abstract

**Background:** Traditional refills of intrathecal infusion pumps rely on manual palpation and the use of external templates, a method that can be challenging in patients with anatomical variations or a high body mass index. Ultrasound guidance has emerged as a precision-based alternative. This study aimed to evaluate the impact of the ultrasound-guided technique versus the conventional template-based technique on patient satisfaction. **Methods:** A quasi-experimental before-and-after study was conducted on a cohort of 45 chronic pain patients. Immediate satisfaction with procedure duration (IPP-SQ), overall treatment efficacy (CRES-4), and pain interference via the Brief Pain Inventory (BPI) were assessed. **Results:** The use of ultrasound was associated with significantly higher satisfaction regarding procedure duration, with a mean score of 5.00 (95% CI: 4.35–5.65) compared to 3.22 (95% CI: 2.70–3.75) with the traditional method (*p* < 0.001). Overall satisfaction (CRES-4) also improved significantly (12.4 vs. 11.3; *p* = 0.001). Regarding patient-reported outcome measures (PROMs), the mean pain intensity in the subsequent week was lower following the ultrasound technique (mean difference −0.48; *p* = 0.040). Technically, no first-attempt failures were recorded under ultrasound guidance in this sample, compared to a 20% re-attempt rate observed with the manual method. **Conclusions:** The transition from the traditional method to ultrasound-guided refill optimizes technical precision and substantially enhances the patient experience. By reducing pain and increasing satisfaction, ultrasound guidance proves to be a valuable resource for improving procedural precision, representing an advancement toward a more personalized medicine approach.

## 1. Introduction

Chronic pain remains a global health challenge, affecting approximately one-third of the adult population and imposing a substantial socio-economic burden [1,2]. In response, the field of precision medicine has shifted pain management toward targeted interventions that prioritize clinical efficacy while limiting systemic toxicity. Among these, intrathecal drug delivery systems represent a cornerstone of personalized therapy for patients refractory to conservative treatments. By delivering medication directly into the subarachnoid space, these implanted pumps achieve potent analgesia with significantly reduced dosages and fewer systemic adverse effects [3,4].

A pivotal element in the success of this individualized therapeutic journey is the maintenance of the device through periodic reservoir refills. Historically, this has been performed using “blind” palpation or standardized external templates. However, such “one-size-fits-all” approaches often fail to account for the unique anatomical landscape of each patient. Clinical factors such as obesity, postsurgical fibrosis, seromas, or device migration create technical barriers that compromise the safety of conventional methods [5]. In these complex scenarios, traditional techniques may lead to multiple needle passes, heightened procedural distress, and life-threatening complications like “pocket fills” (accidental subcutaneous injection) or systemic infections [6].

In this landscape, point-of-care ultrasonography has emerged as a transformative tool for procedural personalization. Unlike blinded methods, ultrasound guidance facilitates real-time visualization of the refill port, allowing the clinician to adapt the needle trajectory to the patient’s specific internal anatomy. Research by Maino et al. [7,8,9] and other authors suggests that this level of precision is particularly advantageous in difficult-access cases or when the pump is implanted at depths exceeding 10 mm [10,11]. Furthermore, the safety profile of the personalized approach is enhanced by the ability to confirm correct needle placement and, if necessary, perform early drug aspiration in the event of an unintended administration [12].

Despite the documented clinical benefits of ultrasonography, there is a notable scarcity of evidence regarding its impact on the humanistic aspect of the procedure. Most current literature prioritizes success rates and procedural duration, often overlooking the patient’s perspective. This oversight is significant, as the repeated nature of reservoir refills means that even minor procedural trauma or anxiety can accumulate over time, potentially leading to treatment burnout or psychological distress. Therefore, an evaluation that integrates patient-reported outcomes is essential to justify the integration of ultrasound as a standard of care in pain clinics [4].

Beyond technical metrics, modern personalized medicine integrates the patient’s subjective experience as a core pillar of quality care. Patient-reported satisfaction is no longer secondary; it is a critical therapeutic marker. A procedure perceived as precise and minimally invasive directly mitigates patient anxiety and fosters long-term adherence to the treatment plan [8]. While the technical superiority of ultrasound is well-documented, there remains a gap in understanding how this technological shift translates into a measurable improvement in patient perception and emotional well-being compared to legacy methods [6].

Building upon this necessity, the present study seeks to provide a comprehensive comparison between ultrasound-guided and template-based refill techniques. We hypothesize that the personalized ultrasound approach will yield superior satisfaction scores by providing a smoother, less invasive experience, thereby reinforcing the role of advanced imaging not just as a technical aid, but as a fundamental tool for improving the quality of life in chronic pain management [11]. Consequently, this study aims to evaluate and compare patient satisfaction levels during intrathecal pump refills, contrasting the personalized ultrasound-guided technique with the conventional template-based approach.

## 2. Methodology

### 2.1. Clinical Context of the Study

This research is defined as a prospective, quasi-experimental study, representing the planned expansion of an ongoing investigative line at the Virgen de las Nieves University Hospital (Granada, Spain). Building upon previous findings that validated the technical precision and analgesic benefits of ultrasound guidance [13], the current phase transitions toward a patient-centric model. The primary goal is to formalize the evaluation of patient satisfaction within a structured framework [14]. This shift from standardized, palpation-based refills to an individualized anatomical approach was implemented as a quality improvement initiative. By utilizing existing hospital resources (on-site ultrasound equipment), this protocol optimizes healthcare delivery and enhances the personalized therapeutic experience without incurring additional institutional costs.

### 2.2. Design

The present study was based on a longitudinal, before-and-after design utilizing a within-patient paired data approach. This methodological framework allowed each participant to serve as their own control—a high-value analytical strategy that effectively neutralized inter-individual confounding variables. Within the context of intrathecal therapies, such control is critical, as it eliminates biases arising from variations in anatomical complexity among subjects, as well as other biological particularities that might otherwise distort the comparison between techniques.

This experimental framework is by no means arbitrary; it aligns closely with the core tenets of personalized medicine. By focusing on the individual’s longitudinal response to different technical interventions, the design captures clinical variability in a manner that cross-sectional studies often overlook.

To ensure maximum scientific rigor, transparency in reporting findings, and data reproducibility, the study was conducted in strict adherence to the STROBE (STrengthening the Reporting of OBservational studies in Epidemiology) statement. These international guidelines represent the gold standard for reporting observational and quasi-experimental research [15].

Ultimately, the selection of this methodological approach addresses a dual imperative: it constitutes the most ethical method, by not depriving patients of potentially beneficial clinical transitions, and the most scientifically sound approach for evaluating the effectiveness and safety of targeted infusion therapies under real-world clinical conditions.

### 2.3. Study Setting and Sampling

Data collection took place at the Chronic Pain Management Unit of the Virgen de las Nieves University Hospital, a specialized center for advanced neuromodulation. The study population consisted of patients with implanted intrathecal devices attending their scheduled maintenance visits. Refill intervals were tailored to each subject’s personalized pharmacological profile, typically ranging from four to twelve weeks. From a total pool of 78 patients, a final cohort of 45 was enrolled, representing 57% of the unit’s target population. All participants completed the full protocol, which involved two sequential refill sessions: an initial procedure using the conventional template-based method, followed by an ultrasound-guided session. This repeated-measures strategy ensures that individual physical characteristics remained constant across both observations, allowing for a precise evaluation of how the change in technique impacted the patient-reported experience.

### 2.4. Inclusion and Exclusion Criteria

Participant recruitment was conducted through the rigorous application of predetermined eligibility criteria, specifically designed to safeguard the robustness and integrity of the comparative analysis. Mandatory inclusion criteria required subjects to be adults (≥18 years) with a fully active and functional intrathecal infusion system at the time of enrollment.

To ensure that the comparison between the template-guided and ultrasound-guided techniques was methodologically equitable and technically consistent, the study exclusively included patients with continuous-flow devices that necessitate the use of standardized refill kits. This instrumental homogeneity served to mitigate external technical variability, thereby ensuring that any observed differences were attributable solely to the method of localization and access to the refill port.

The ethical process commenced with a detailed explanation of the study to each candidate, culminating in the acquisition of written informed consent. In strict compliance with international bioethical standards and data protection regulations, each participant was assigned a unique alphanumeric code. This pseudonymization system was fundamental to guaranteeing the complete anonymization of clinical and personal information, thus maintaining the highest standards of integrity and confidentiality throughout the longitudinal follow-up phase and subsequent data processing.

### 2.5. Technical Procedures and Interventions

In the conventional phase (control), the refill followed the manufacturer’s instructions, relying on manual palpation of the device’s silhouette. Once identified, a sterile template was positioned on the skin to estimate the location of the access port, and the puncture was performed perpendicularly through the guide [16].

In the personalized phase (ultrasound-guided), point-of-care ultrasonography allowed for the real-time mapping of each patient’s internal anatomy. The refill port was identified as an anechoic vertical signal within the hyperechoic metallic casing of the pump. This allowed the clinician to mark the exact center of the reservoir, adapting the entry point to the device’s specific depth and orientation. In cases of significant anatomical distortion or device rotation, ultrasound served as a vital safety tool, facilitating successful access even when traditional landmarks were obscured [17].

### 2.6. Data Collection and Variable Management

The study utilized a prospective data collection notebook to record variables essential for precision healthcare, including age, sex, and specific medication (e.g., morphine vs. ziconotide). To assess the sensory and functional impact of the procedures, the validated Brief Pain Inventory (BPI) was used [18], measuring pain intensity and its interference with daily life. Patients were reassessed one week post-procedure to capture the full scope of their recovery.

Furthermore, global satisfaction was quantified using the CRES-4 Scale (Consumer Reports Effectiveness Scale) [19]. This instrument provided a standardized metric (0–100) of the perceived effectiveness and emotional benefit of the ultrasound-guided transition. This multidimensional approach—combining clinical metrics with Patient-Reported Outcome Measures (PROMs)—ensures that the findings reflect a truly holistic and personalized assessment of the care provided. The data collection period for this study was between March 2024 and September 2025.

### 2.7. Statistical Analysis

For within-participant comparisons of outcomes assessed after the traditional and ultrasound-guided procedures, we fitted generalized linear mixed-effects models with a participant-specific random intercept to account for repeated measurements. The conditional distribution and link function were selected according to the empirical distribution of each outcome, using Gaussian, Poisson, or negative binomial models as appropriate. Treatment technique was included as a fixed effect. Adjusted comparisons between techniques were summarized using model-based marginal means and mean differences with 95% confidence intervals, estimated from the fitted models with the marginaleffects package. All analyses were conducted in R software v4.5.2.

### 2.8. Ethical Considerations and Data Protection

The study was conducted in accordance with the Declaration of Helsinki and received formal approval from the Provincial Research Ethics Committee of Granada on 30 June 2022 (Ref: PEIBA_SEG 6 PLANT22052710190). All participants provided written informed consent after being fully briefed on the study’s observational nature. This study was categorized as a quasi-experimental, non-interventional project within routine clinical practice, and therefore did not require prospective clinical trial registration under Spanish RD 1090/2015. Data protection was strictly maintained through encrypted systems and anonymized coding, in full compliance with current European and national privacy regulations.

## 3. Results

A total of 45 participants were included (20 women and 25 men), with a median age of 56 years (interquartile range, 51 to 64). Most participants received intrathecal morphine (84.4%), and the median interval between procedures was 72 days (49 to 84). The most common indications were lumbar pain or spinal degenerative disease and postsurgical spinal pain; the remaining baseline characteristics are summarized in Table 1.

**Figure 1:**
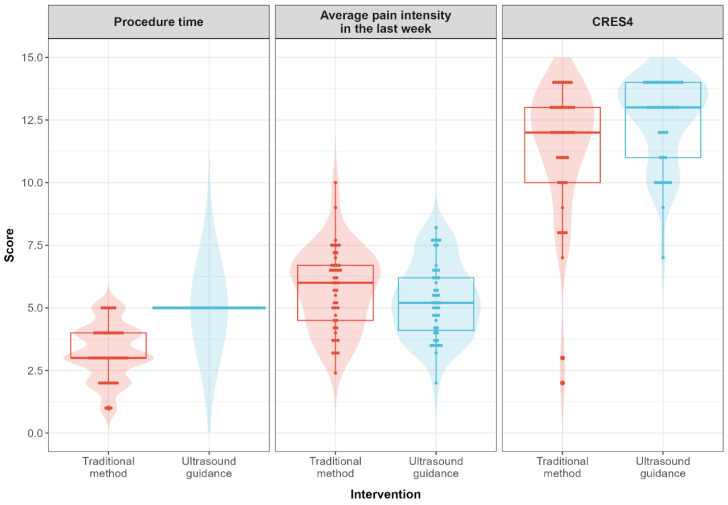
Distribution of patient-reported outcomes after traditional versus ultrasound-guided intrathecal pump refill procedures.

Ultrasound guidance was associated with higher immediate post-procedure satisfaction regarding procedure duration, with a predicted mean IPP-SQ time score of 5.00 (95% CI, 4.35 to 5.65) compared with 3.22 (95% CI, 2.70 to 3.75) for the traditional method (mean difference, 1.78; 95% CI, 0.94 to 2.62; *p* < 0.001). Overall satisfaction with the treatment received (CRES-4) was also higher after the ultrasound-guided procedure (12.4 [95% CI, 11.8 to 13.1] vs 11.3 [95% CI, 10.7 to 12.0]; mean difference, 1.11 [95% CI, 0.45 to 1.78]; *p* = 0.001). Average pain intensity during the subsequent week was modestly lower following ultrasound guidance (mean difference, −0.48; 95% CI, −0.94 to −0.02; *p* = 0.040). The remaining comparisons, including perceived procedural safety, perceived professional expertise, and pain interference, are reported in Table 2 and Figure 1.

As shown in Figure 1, the distributions of the patient-reported outcomes for the two techniques overlapped considerably, particularly for average pain intensity and pain interference on the BPI. The between-group separation was most apparent for procedure duration satisfaction, whereas for pain outcomes the distributions shared most of their range, with a shift in central tendency rather than a clear displacement. These visual patterns are consistent with the modest effect sizes obtained in the mixed-effects models and indicate that, although mean differences reached statistical significance, a substantial proportion of participants reported similar pain scores with both techniques.

## 4. Discussion

The present study demonstrates that the use of ultrasound guidance in refilling intrathecal infusion pumps represents a qualitative advance in precision medicine by transforming a routine technical procedure into an intervention centered on the patient experience. Our findings confirm that direct visualization of the device not only optimizes the safety and time parameters observed in previous phases of this research but also positively impacts multidimensional satisfaction and the subject’s well-being—key elements for adherence and the success of personalized analgesic therapies.

Historically, the traditional template-based method has been the standard; however, this approach ignores individual anatomical variability. Our results reveal that ultrasound guidance is associated with significantly higher immediate satisfaction regarding process duration (score of 5.00 versus 3.22; *p* < 0.001). This perception of efficiency by the patient is complemented by an improvement in overall treatment satisfaction (CRES-4), where the ultrasound-guided technique outperformed the conventional method (12.4 vs. 11.3; *p* = 0.001). This difference, though modest in scale, is clinically relevant in the context of personalized medicine: by adapting the technique to the patient’s unique anatomy (especially in cases of obesity or fibrosis), procedural uncertainty and stress are reduced, enhancing user confidence in the healthcare system.

The magnitude of the observed between-technique differences, however, requires careful interpretation. The mean reduction in BPI average pain intensity was −0.48 points on a 0 to 10 numerical scale, which falls below the minimal clinically important difference (MCID) proposed by the IMMPACT consensus for chronic pain trials, where reductions of approximately 1 to 2 points or 10 to 20% from baseline are required to be considered clinically meaningful [20]. A similar consideration applies to the CRES-4 scale, for which a validated MCID has not been established and for which the absolute between-technique difference was of modest magnitude. Statistical significance in this context should therefore not be conflated with clinical relevance. The most robust finding of the present study is the improvement in procedure duration satisfaction (IPP-SQ), where the mean difference of 1.78 points on a 0 to 6 anchored scale reflects a consistent patient-level perception of a shorter and less burdensome procedure, together with the near-complete elimination of repeat punctures.

In any case, the consistency of these data with the scientific literature reinforces the validity of the paradigm shift. While previous studies, such as that by Maino et al. [7], focused on success rates, and others, such as Stone et al. [6], found no significant temporal differences, our research demonstrates that technical precision directly translates into pain reduction. The observed reduction in pain intensity one week after the procedure suggests a clinical benefit associated with decreased tissue trauma. By avoiding multiple puncture attempts and excessive port manipulation, the local inflammatory response is minimized. Therefore, these findings likely reflect a decrease in procedure-related pain rather than a shift in the patient’s underlying pathology. In fact, we observed that mean pain intensity during the subsequent week was lower in the ultrasound-guided group (*p* = 0.040), suggesting that avoiding multiple punctures and tissue trauma has a residual benefit on the patient’s quality of life. This finding supports the conclusions of Singa et al. [7] regarding patient comfort and underscores that ultrasound mitigates critical risks such as pocket fill or erroneous puncture, factors that are often sources of procedural anxiety [21,22,23].

From the perspective of management and advanced clinical practice, the implementation of this model, led by specialized nursing in high-volume pain units, optimizes workflows without increasing direct costs [24]. The capacity of nursing staff to perform refills with greater autonomy and precision not only improves system efficiency, potentially reducing waiting lists, but also raises the standard of safety [25]. By placing the patient at the center, ultrasound guidance meets the requirements of modern health interventions: being safe, effective, and highly satisfactory for the user [26].

Fundamentally, these findings underscore that the transition to ultrasound-guided procedures constitutes a paradigm shift toward truly personalized healthcare. By shifting from a standardized, one-size-fits-all approach to an individualized anatomical mapping strategy, we address the unique physiological variables of each patient. This precision-based methodology ensures that the therapeutic intervention is tailored to the patient’s specific internal landscape, thereby minimizing procedural distress and optimizing clinical outcomes. Consequently, this study demonstrates that personalizing technical maneuvers is as critical to long-term success as the pharmacological regimen itself, reinforcing the patient-provider bond and elevating the standard of chronic pain management.

Despite the strength of the evidence obtained in a real-world clinical setting, this study has certain limitations. First, its monocentric and quasi-experimental design may limit the generalizability of the findings. A randomized crossover approach was considered but deemed unfeasible due to clinical and ethical constraints; intrathecal refill intervals are strictly dictated by individual pharmacological requirements, and shortening them for randomization would risk therapy interruption or under-dosing. Furthermore, as ultrasound was implemented as an institutional quality-improvement initiative, maintaining the template-based procedure in a patient subgroup following the protocol change was considered ethically questionable, given prior evidence of lower first-attempt success rates and a higher risk of complications with the conventional approach.

Moreover, while our sample represents a significant proportion of the patients treated at our center, the total size (n = 45) and the monocentric, non-randomized nature of the study limit the immediate extrapolation of the results. Although the findings strongly support the benefits of ultrasound, they should be considered exploratory. Further multicenter studies with larger cohorts are required to definitively establish ultrasound guidance as the new clinical gold standard in intrathecal therapy.

Another relevant limitation is that patients were not stratified according to body mass index or device depth, factors that could potentially influence the agility of the conventional technique. In patients with favorable anatomy and easily palpable ports, the template-guided method might prove faster by bypassing ultrasound setup time, although in our overall cohort, the precision of ultrasound compensated for any temporal differences. However, all procedures were performed by the same team of specialized nurses with uniform expertise in intrathecal refills, which helps mitigate operator-related variability.

This study constitutes a starting point for the development of future research lines that delve deeper into the transition toward precision medicine within the field of neuromodulation. One of the primary derived research avenues involves conducting multicenter randomized clinical trials to establish ultrasound guidance as the definitive gold standard, thereby overcoming the limitations of monocentric designs. Likewise, it is imperative to explore the impact of this technique in patient subgroups with complex anthropometric profiles, specifically those with a high body mass index or devices implanted at depths exceeding 10 mm, where the conventional method may exhibit higher failure rates. Other areas of significant interest include long-term economic impact analysis by evaluating the reduction in serious complications, such as accidental subcutaneous pocket fills, and measuring the learning curve required for nursing staff to achieve technical excellence.

Regarding the practical applications of these findings, the integration of ultrasonography into chronic pain units could facilitate a progressive optimization of workflows and contribute to reinforcing patient safety [7]. The ability to visualize the refill port in real time facilitates a personalized approach that adapts to the unique internal anatomy of each individual, minimizing tissue trauma and procedural stress. This technical precision not only translates into greater user satisfaction regarding process efficiency but also serves as a protective factor against adverse events, elevating the standard of care provided by specialized nursing [19]. Ultimately, the systematic implementation of this protocol promotes the humanization of healthcare by positioning the patient’s subjective experience and physical well-being as central pillars of the therapeutic approach in complex pain management.

## 5. Conclusions

The integration of ultrasound guidance into the intrathecal infusion pump refill procedure suggests a valuable advancement toward a patient-centered model of precision medicine. The findings of this study indicate that the use of ultrasound can outperform the traditional template-based method in this cohort, achieving an improvement in overall user satisfaction and a reduction in perceived pain intensity. This approach facilitates a shift toward personalized medicine, where the technique is adapted to the unique anatomical environment of each patient, allowing for an intervention tailored to individual needs.

By providing direct, real-time visualization of the refill port, this technique optimized first-attempt success in our sample, reducing the uncertainty of manual palpation. Furthermore, the implementation of this protocol by specialized nursing staff, following standardized training, represents an efficient advanced practice strategy. Ultimately, the ultrasound-guided technique shows potential to optimize clinical safety and humanize the care of chronic patients; therefore, its adoption merits consideration as a highly precise alternative in intrathecal therapy units.

## Figures and Tables

**Table 1:** Baseline characteristics of the study participants.

Characteristic	Female N = 20 ^a^	Male N = 25 ^a^	Overall N = 45 ^a^
Age, years	58 (50, 65)	56 (51, 61)	56 (51, 64)
Medical history			
Postsurgical spinal pain	4 (20.0%)	9 (36.0%)	13 (28.9%)
Lumbar pain/spinal degenerative disease	6 (30.0%)	10 (40.0%)	16 (35.6%)
Chronic widespread musculoskeletal pain	7 (35.0%)	4 (16.0%)	11 (24.4%)
Structural musculoskeletal abnormalities	1 (5.0%)	0 (0.0%)	1 (2.2%)
Tumors/Rare diseases	2 (10.0%)	1 (4.0%)	3 (6.7%)
Other	0 (0.0%)	1 (4.0%)	1 (2.2%)
Treatment			
Ziconotide	1 (5.0%)	6 (24.0%)	7 (15.6%)
Morphine	19 (95.0%)	19 (76.0%)	38 (84.4%)
Time between procedures, days	74 (60, 84)	64 (38, 80)	72 (49, 84)

^a^ Median (Q1, Q3); n (%).

**Table 2:** Predicted mean patient-reported outcomes after traditional versus ultrasound-guided intrathecal pump refill procedures.

Parameter	Traditional Method ^a^	Ultrasound Guidance ^a^	Difference (95% CI)	*p*
IPP-SQ				
Procedure duration	3.22 (2.70–3.75)	5.00 (4.35–5.65)	1.78 (0.94–2.62)	<0.001
Procedure safety	4.18 (3.59–4.77)	5.00 (4.35–5.65)	0.82 (−0.06–1.71)	0.069
Professional expertise	4.89 (4.24–5.53)	5.00 (4.35–5.65)	0.11 (−0.81–1.03)	0.813
BPI				
Average pain intensity in the last week ^b^	5.76 (5.31–6.21)	5.28 (4.83–5.73)	−0.48 (−0.94–−0.02)	0.040
Interference of pain in daily life	5.72 (5.16–6.28)	5.52 (4.96–6.08)	−0.20 (−0.73–−0.33)	0.463
CRES4				
Satisfaction with the treatment received	11.3 (10.7–12.0)	12.4 (11.8–13.1)	1.11 (0.45–1.78)	0.001

^a^ Predicted mean (95% CI). ^b^ Measured 1 week after intervention. Abbreviations: IPP-SQ, Immediate Post-Procedure Satisfaction Questionnaire; BPI (Brief Pain Inventory); CRES4 (Consumer Reports Effectiveness Scale).

## Data Availability

Data regarding this study is available upon request to the corresponding author.
